# Ultrasound-guided central venous catheter placement: a structured review and recommendations for clinical practice

**DOI:** 10.1186/s13054-017-1814-y

**Published:** 2017-08-28

**Authors:** Bernd Saugel, Thomas W. L. Scheeren, Jean-Louis Teboul

**Affiliations:** 1 Department of Anesthesiology, Center of Anesthesiology and Intensive Care Medicine, University Medical Center Hamburg-Eppendorf, Martinistrasse 52, 20246 Hamburg, Germany; 2 Department of Anesthesiology, University of Groningen, University Medical Centre Groningen, Groningen, The Netherlands; 3 Service de Réanimation Médicale Hôpital de Bicêtre, Hôpitaux Universitaires Paris-Sud, AP-HP, Le Kremlin-Bicêtre, France

**Keywords:** Central venous access, Ultrasound, Internal jugular vein, Subclavian vein, Femoral vein, Short axis, Long axis, Out of plane, In plane

## Abstract

The use of ultrasound (US) has been proposed to reduce the number of complications and to increase the safety and quality of central venous catheter (CVC) placement. In this review, we describe the rationale for the use of US during CVC placement, the basic principles of this technique, and the current evidence and existing guidelines for its use. In addition, we recommend a structured approach for US-guided central venous access for clinical practice. Static and real-time US can be used to visualize the anatomy and patency of the target vein in a short-axis and a long-axis view. US-guided needle advancement can be performed in an "out-of-plane" and an "in-plane" technique. There is clear evidence that US offers gains in safety and quality during CVC placement in the internal jugular vein. For the subclavian and femoral veins, US offers small gains in safety and quality. Based on the available evidence from clinical studies, several guidelines from medical societies strongly recommend the use of US for CVC placement in the internal jugular vein. Data from survey studies show that there is still a gap between the existing evidence and guidelines and the use of US in clinical practice. For clinical practice, we recommend a six-step systematic approach for US-guided central venous access that includes assessing the target vein (anatomy and vessel localization, vessel patency), using real-time US guidance for puncture of the vein, and confirming the correct needle, wire, and catheter position in the vein. To achieve the best skill level for CVC placement the knowledge from anatomic landmark techniques and the knowledge from US-guided CVC placement need to be combined and integrated.

## Background

Although placement of a central venous catheter (CVC) is a routine procedure in intensive care medicine and anesthesiology, acute severe complications (such as arterial puncture or cannulation, hematoma, hemothorax, or pneumothorax) occur in a relevant proportion of patients [1, 2]. The use of ultrasound (US) has been proposed to reduce the number of CVC complications and to increase the safety and quality of CVC placement. In this review, we describe the rationale for the use of US during CVC placement, the basic principles of this technique, and the current evidence and existing guidelines for its use. In addition, we recommend a structured approach for US-guided central venous access for clinical practice.

## Rationale for ultrasound-guided central venous catheter placement

Traditionally, CVC placement is performed using landmark techniques based on the knowledge of anatomic structures and palpation of arteries next to the veins. These landmark techniques cannot account for anatomic variations at the CVC insertion site. Anatomic variations to the "normal anatomy", however, have been described in a relevant proportion of patients for the internal jugular vein (IJV), the subclavian vein (SV), and the femoral vein (FV) [3–11]. In addition to anatomic variations, venous thrombosis that is especially common in oncologic and critically ill patients can make CVC placement impossible or dangerous for the patient [9].

The described anatomic variations and the presence of venous thrombosis can hardly be identified using a landmark technique. In contrast, US can be used to easily visualize anatomic structures and confirm patency of the vein and thus help to avoid unintended arterial puncture or unsuccessful cannulation. In addition, US can facilitate CVC placement in special clinical situations in which landmark techniques based on palpation of the arterial pulse are challenging or impossible (e.g., femoral CVC placement during cardiopulmonary resuscitation [12] or in patients with a nonpulsatile ventricular assist device).

## Ultrasound for central venous catheter placement: basic principles and techniques

### Ultrasound probe

US probes best suited for CVC placement are small linear array probes with high-frequency transducers (5–15 MHz) [13]. These probes usually have a scanning surface of about 20–50 mm and allow high-resolution imaging of superficial anatomic structures [13]. 2D imaging (complemented by Doppler US functions) is currently the standard technique used for US-guided central venous access [13]. All US probes have an index mark (a small physical notch on one side of the probe) that corresponds with an orientation marker on one side of the US scan sector shown on the US device screen and thus helps to obtain the correct probe orientation during US examination. Preferably, US machines should have the ability to record and save US images and loops for clinical documentation (and teaching purposes) [13].

### Ultrasound techniques for central venous catheter placement

US can be used in different ways to facilitate CVC placement. "Static" US (also called indirect US) describes a technique using US only before CVC placement to identify the anatomy of the target vein and adjacent anatomic structures (including the patency of the vein and its dimensions and depth from the skin) [14]. This approach of preprocedural US evaluation is also referred to as "US-assisted" CVC placement.

In contrast, "real-time" US (also called direct US) describes a technique of needle advancement and vessel puncture under permanent US control (i.e., the needle is permanently visualized on the US screen). This is also referred to as "US guidance" [14].

### Short-axis/long-axis and out-of-plane/in-plane views

The US probe can be placed in a transverse position relative to the vessel, resulting in a "short-axis" view on the US screen (i.e., a cross-sectional image of the vessel). A "long-axis" view (i.e., a longitudinal image of the vessel) is obtained by placing the US probe in a parallel position relative to the course of the vessel. Short-axis and long-axis views can be used for both US assistance and guidance of CVC placement. Of note, the terms "out-of-plane" and "in-plane" describe the direction of the needle relative to the US plane, refer to US-guided needle advancement, and should not be mixed up with the terms "short-axis" and "long-axis".

**Fig. 1:**
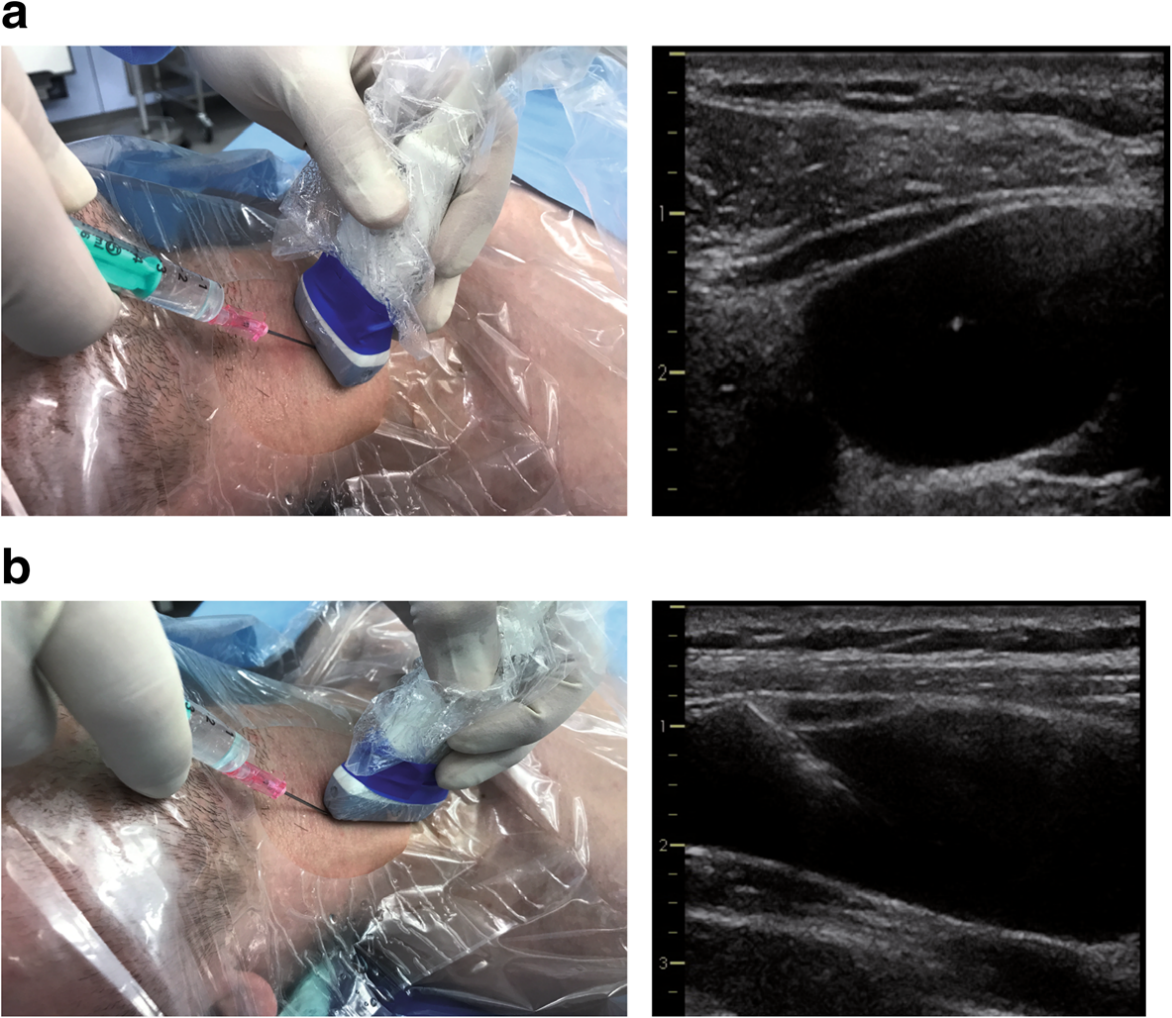
Ultrasound probe orientation and view of the needle. Ultrasound guidance during needle advancement can be performed using a short-axis probe orientation and an out-of-plane view of the needle (**a**) or a long-axis probe orientation and an in-plane view of the needle (**b**)

For real-time US guidance, different US approaches can be used. US guidance during needle advancement can be performed using: a short-axis probe orientation and an out-of-plane view of the needle (Fig. 1a); a long-axis probe orientation and an in-plane view of the needle (Fig. 1b); or a so-called oblique orientation [15]. It is important to understand that the user needs to align the US plane and the needle plane containing the needle that appears on the screen as a point (short-axis/out-of-plane) or an echogenic line (long-axis/in-plane) with ring-down artifacts [14].

Whether or not one approach is superior to the other cannot be answered rigorously based on the existing data. The advantage of the short-axis/out-of-plane view is that it allows better visualization of the vein in relation to the artery and other anatomic structures, and thus might more sufficiently help to avoid accidental arterial puncture [15]. The short-axis/out-of-plane approach is easier to learn for physicians not familiar with US [16]. Among experienced US users, the short-axis/out-of-plane approach seems to result in a higher success rate with the first attempt for CVC placement in the IJV and SV [17, 18]. However, in the short-axis view, the needle is only visualized as an echogenic point (that must not necessarily be the tip of the needle). In contrast, when using the long-axis/in-plane view, the entire needle in its complete course and the depth of the needle tip can be visualized on the US image, thus reducing the risk of penetration of the posterior vessel wall [15, 19].

Combining advantages of both techniques, the oblique axis view (a view that is halfway between the short-axis and the long-axis view with the US probe placed at approximately 45° with respect to the target vessel) can be used by experienced US users [20, 21].

### Can ultrasound make central venous catheter placement safer? What is the evidence?

The use of US to reduce the number of complications related to vascular access for CVC placement has been evaluated in numerous previous studies in a variety of clinical settings. Recent Cochrane systematic reviews and meta-analyses summarize the current evidence for US guidance versus anatomic landmark techniques for CVC placement in the IJV [22], SV [23], and FV [23] with regard to complications of CVC placement. These meta-analyses included adult and pediatric patients treated in the intensive care unit or the operating room and compared conventional landmark techniques with techniques using static or real-time US or Doppler US. The primary outcome measure was the total rate of peri-interventional complications and adverse events.

For the IJV, 35 trials enrolling a total of 5108 patients were included in the meta-analysis [22]. The analysis demonstrated that the use of US for CVC placement in the IJV reduces the total rate of complications compared with conventional landmark techniques (US, 48 complications in 1212 patients (4.0%) vs landmark, 161/1194 (13.5%); risk ratio (95% confidence interval (CI)) 0.29 (0.17–0.52)). The overall success rate was higher when US was used (US, 2120/2172 (97.6%) vs landmark, 1900/2168 (87.6%); risk ratio (95% CI) 1.12 (1.08–1.17)) [22]. In addition, the use of US resulted in a decrease in the rate of arterial puncture, hematoma formation, and number of attempts and time until successful cannulation, and in an increase in the success rate with the first attempt of puncture [22]. The benefits of US-guided or US-assisted CVC placement with regard to the total complication rate, overall success rate, and number of attempts until success were consistent across experienced and inexperienced operators. Thus, this meta-analysis clearly provides evidence that US offers gains in safety and quality during CVC placement in the IJV. The quality of the evidence, however, was very low for most outcome measures and the heterogeneity among the studies was high.

For the SV, a meta-analysis including nine studies with 2030 patients showed that the use of US resulted in a reduced rate of accidental arterial puncture (US, 2/242 (0.8%) vs landmark, 15/256 (5.9%); risk ratio (95% CI) 0.21 (0.06–0.82)) and hematoma formation (US, 3/242 (1.2%) vs landmark, 17/256 (6.6%); risk ratio (95% CI) 0.26 (0.09–0.76)) [23]. However, no statistically significant difference was found between the use of US and the conventional landmark technique with regard to the total complication rate, the overall success rate, the number of attempts until success, the time to successful cannulation, and the success rate with the first attempt [23].

For CVC placement in the FV, the use of US compared with the landmark technique increased the overall success rate (US, 134/150 (89.0%) vs landmark, 127/161 (78.9%); risk ratio (95% CI) 1.11 (1.00–1.23)) and the success rate with the first attempt (US, 91/107 (85.0%) vs landmark, 57/117 (48.7%); risk ratio (95% CI) 1.73 (1.34–2.22)) [23].

Although the use of US offers small gains in safety and quality, the authors conclude that the meta-analysis does not generally support the use of US for CVC placement in the SV and FV [23].

On behalf of the Canadian Perioperative Anesthesia Clinical Trials Group, Lalu et al. [24] performed a systematic review and meta-analysis of US-guided SV catheterization. Based on data from 10 studies (including 2168 patients; six real-time US studies, one static US study, three Doppler US studies), the authors revealed that US reduced the overall complication rate compared with the landmark technique (odds ratio (95% CI) 0.53 (0.41–0.69)). Real-time US particularly reduced accidental arterial puncture, pneumothorax, and hematoma formation.

A CVC via the SV can be placed using either an infraclavicular (most commonly used) or a supraclavicular approach. To the best of our knowledge there are no randomized controlled trials on US-guided CVC placement via the SV comparing the supraclavicular and the infraclavicular approach. The supraclavicular approach (using different US probes) needs to be evaluated in future studies.

When discussing the evidence for US during CVC placement at the different anatomic sites based on the available studies and meta-analyses, one needs to keep in mind that—compared to the IJV—it might be more challenging to prove the advantages of US for CVC placement in the SV, because the ultrasound approach is technically more challenging, and in the FV, because severe complications other than arterial puncture occur infrequently.

## Guidelines for ultrasound-guided central venous catheter placement

Various recommendations and guidelines with different clinical scopes and for different target audiences have been published during the last years.

In 2012, a joint guideline from the American Society of Echocardiography and the Society of Cardiovascular Anesthesiologists [15] strongly recommended the use of real-time US for CVC placement in the IJV (category A, level 1 evidence), while it was not recommended for the SV (category A, level 3 evidence). For the FV, no recommendation for routine use of US was made because of insufficient scientific evidence (category C, level 2 evidence).

A practice guideline from the American Society of Anesthesiologists task force, also in 2012 [25], recommended the use of static US imaging in elective situations for prepuncture identification of the anatomy and to evaluate the vessel localization and patency and real-time US for venipuncture for the IJV. Further, it is recommended that both static and real-time US "may" be used for CVC placement in the SV or FV [25].

For CVC placement in critically ill patients treated in the intensive care unit, an international expert panel recommended in 2012 the routine use of US for short-term and long-term central venous access in adults [13]. More specifically, the panel recommended the utilization of 2D US imaging with a long-axis/in-plane technique for vascular access [13] and agreed on the very strong recommendation (based on Level A evidence) that "US-guided vascular access has to be used because it results in clinical benefits and reduced overall costs of care makes it cost-effective” [13].

The guidelines for the appropriate use of bedside general and cardiac US from the American College of Critical Care Medicine [26] give a strong (1-A) recommendation for the general use of US for central venous access in real-time technique (1-B) using a short-axis approach (1-B). Regarding the site for CVC placement, the guidelines give a strong (1-A) recommendation for the IJV and the FV, but a conditional recommendation (2-C) for the SV [26].

A guideline from the European Federation of Societies for Ultrasound in Medicine and Biology (EFSUMB) [9] also recommends pre-interventional US vessel screening of target vessels to determine the most appropriate anatomical site and the optimal patient position (5-D) and routine real-time US guidance during CVC placement (1-A) [9].

In 2016, the Association of Anaesthetists of Great Britain and Ireland [27] also recommended the routine use of US for CVC placement in the IJV. In addition, the expert group recommends US use "for all other central venous access sites, but recognizes evidence is, at present, limited" [27]. Nevertheless, the recommendation also underlines that the understanding of the landmark technique is necessary for situations when US is not available.

## Use of ultrasound for central venous catheter placement in clinical practice

Several survey studies evaluated the attitudes and beliefs of intensivists and anesthesiologists on the use of US for CVC placement and the frequency of its use in clinical practice.

In 2008, McGrattan et al. [28] performed a survey among 2000 senior anesthesiologists in the United Kingdom and revealed that only 27% of these stated using US as the first-choice approach for CVC placement in the IJV (50% used the surface landmark technique and 30% palpation of the carotid artery as first-choice approaches).

Among emergency physicians in the United States, 44% stated in 2014 that they never use US to guide CVC placement [10]. On the other hand, 20% and 9% of respondents stated using US in at least 90% and 100% of cases, respectively.

A survey among 784 intensivists in the United States performed in 2016 [29] revealed a moderate to very frequent use of US depending on the site for CVC placement ranging from 31% for the SV to 80% for the IJV (45% for the FV). Barriers to the use of US reported by these respondents were limited availability of US equipment (28%), perception of increased time for US-guided CVC insertion (22%), and concerns about losing skills for the landmark technique (13%) [29].

Among 190 French intensivists, a practice survey [30] reported high rates of US use for CVC placement in 2016, with 18% and 50% of physicians always or almost always, respectively, using an US-guided CVC technique (6% never, 10% almost never, 17% half of the time). Interestingly, a higher proportion of residents compared with senior doctors stated always or at least almost always using US.

## How to perform ultrasound-guided central venous catheter placement? Recommendations for clinical practice—a systematic approach

For clinical practice, we recommend a systematic approach including the following steps:

I. Identify anatomy of the insertion site and localization of the vein.
II. Confirm patency of the vein.
III. Use real-time US guidance for puncture of the vein.
IV. Confirm needle position in the vein.
V. Confirm wire position in the vein.
VI. Confirm catheter position in the vein.

### Identify anatomy of the insertion site and localization of the vein

As a first step, one should use US to identify the anatomy of the insertion site (vein and artery, adjacent anatomic structures) and the localization of the target vein. This includes checking for anatomic variations of the vessels (both vein and artery) and the localization of the vein in relation to the artery. This step requires combining a profound knowledge about anatomic structures and landmarks with the competencies required for US-guided CVC placement (such as knowledge about probe orientation and image display, converting the 2D US image into 3D reality, and hand–eye coordination) [31]. Given the variability in anatomic structures, this first step of US assessment is best performed before prepping and draping of the puncture site and the US probe.

**Fig. 2:**
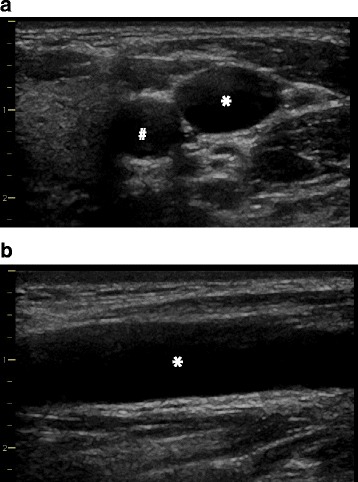
Ultrasound views to identify the anatomy of the target vein. Short-axis (transverse) view (**a**) and long-axis (longitudinal) view (**b**) of the right internal jugular vein (*) and its anatomic relation to the carotid artery (#)

**Fig. 3:**
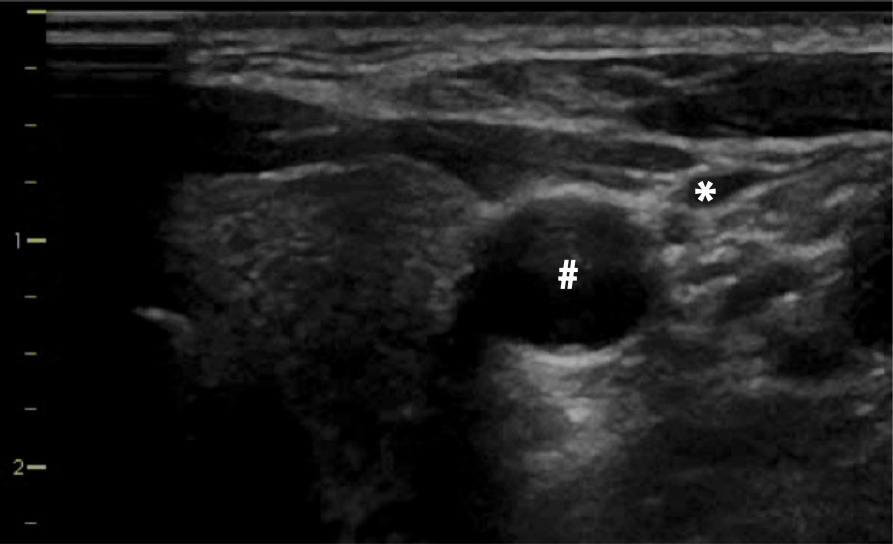
Ultrasound view of a small internal jugular vein. Short-axis (transverse) view of a small right internal jugular vein (*) and its anatomic relation to the carotid artery (#) (e.g., in a patient with intravascular hypovolemia)

**Fig. 4:**
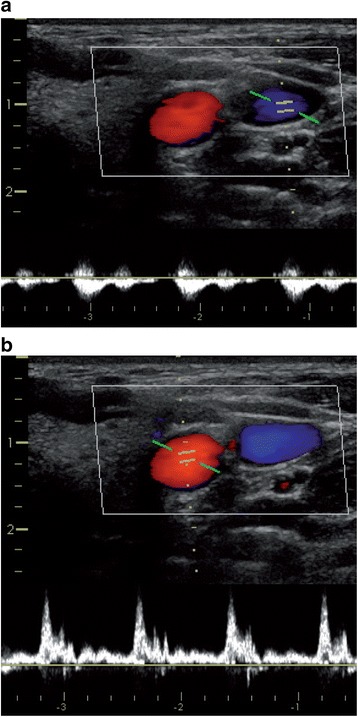
Color Doppler imaging and Doppler flow measurements. Short-axis (transverse) view of the right internal jugular vein (blue) and the carotid artery (red) using color Doppler imaging and Doppler flow measurements of the venous (**a**) and arterial (**b**) blood flow profile (Color figure online)

The location of the vein and its anatomic relation to the artery is best identified when using both a short-axis (transverse) and a long-axis (longitudinal) view of the vessels (Fig. 2a, b). This also allows identifying hypoplastic veins or underfilling of the veins due to intravascular hypovolemia (Fig. 3). To exactly differentiate between venous and arterial vessels one can additionally perform color Doppler imaging and apply Doppler flow measurements to derive venous and arterial Doppler flow profiles (Fig. 4a, b).

### Confirm patency of the vein

By applying pressure to the vein and thus testing its compressibility with the US probe, one can confirm the patency of the vein and thus exclude venous thrombosis. Of note, in patients with very low arterial blood pressure (systolic arterial pressure < 60 mmHg), the artery might also be compressible [14].

To further confirm the patency of the vein and to quantify venous and arterial blood flow, color Doppler imaging and Doppler flow measurements should be performed (Fig. 4a, b).

### Use real-time ultrasound guidance for puncture of the vein

CVC placement should be performed using US guidance. This requires an aseptic approach to avoid catheter-related bloodstream infections. An aseptic technique includes: prepping and covering the puncture site with a large sterile drape; wearing a hat, a mask, sterile gloves, and a sterile body gown; covering the US probe and cable with a sterile cover/shield; and using a sterile conductive medium (US gel) [13, 32].

The position of the operator performing US-guided CVC placement should be such that he/she has the insertion site, the needle, and the US screen in their line of sight during needle insertion [13]. Usually, the operator should hold the US probe with the nondominant hand while advancing the needle with the dominant hand. This approach is referred to as the "single-operator technique" and allows the operator to optimally align the US plane and the direction of the needle.

**Fig. 5:**
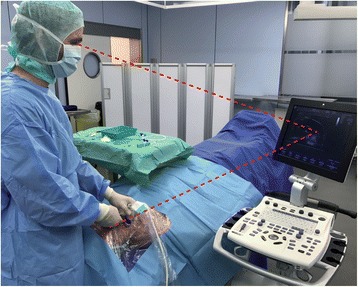
Practical aspects of ultrasound-guided central venous catheter placement in the internal jugular vein using the "single-operator technique”. An aseptic approach including covering the puncture site with a large sterile drape, using sterile barriers (hat, mask, sterile gloves, sterile body gown), and covering the ultrasound probe and cable with a sterile cover is shown. The position of the operator (who holds the ultrasound probe with the nondominant hand while advancing the needle with the dominant hand) allows aligning the insertion site, the needle, and the ultrasound screen in the line of sight during needle insertion (red lines) (Color figure online)

These practical aspects of US-guided CVC placement are illustrated in Fig. 5.

While advancing the needle, its tip should be constantly identified with US during the needle approach to the vein and puncture of the vein. This can be done using a short-axis/out-of-plane view or a long-axis/in-plane view.

### Confirm needle position in the vein

**Fig. 6:**
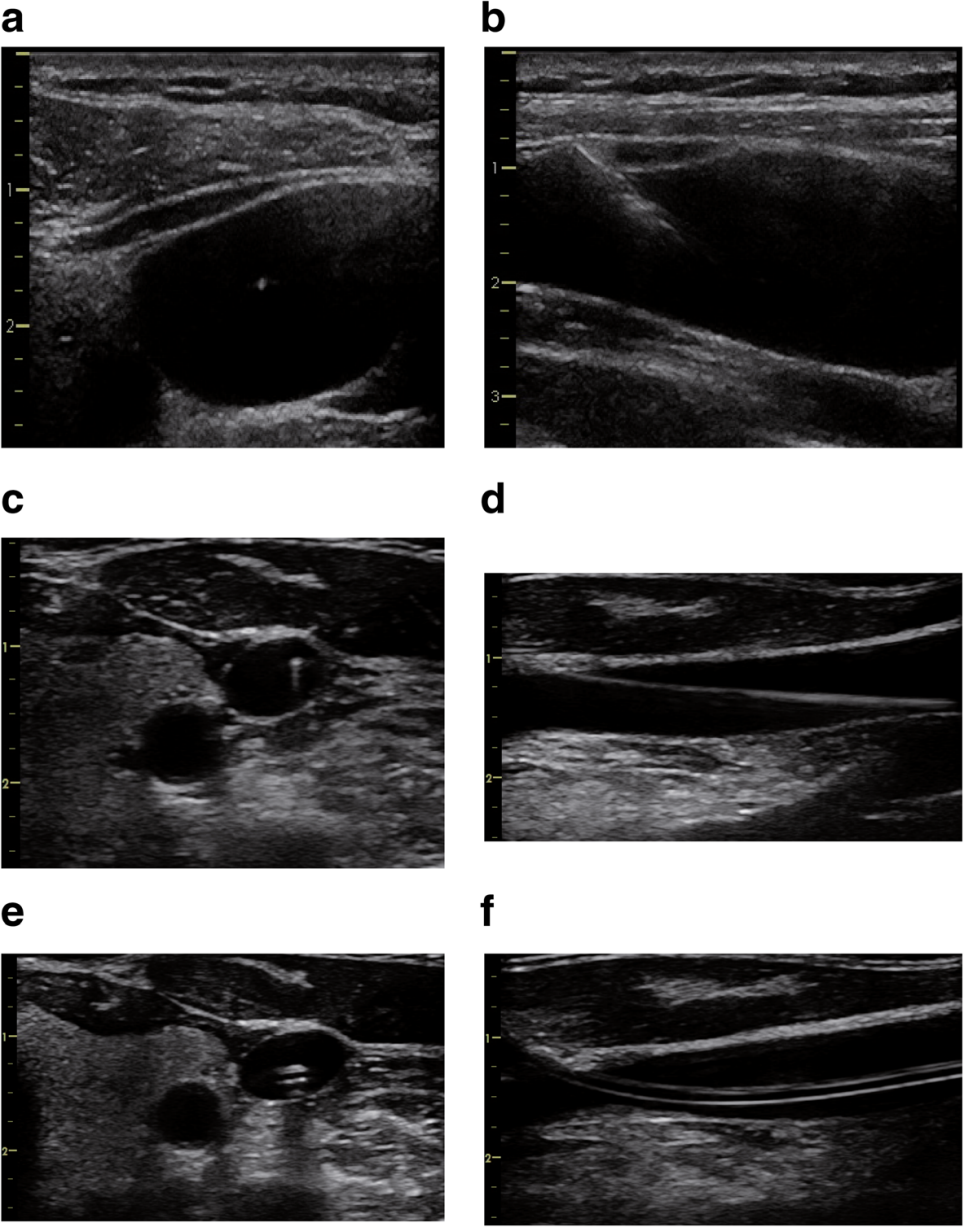
Ultrasound to confirm needle, wire, and catheter position in the vein. Ultrasound images during real-time ultrasound-guided central venous catheter placement in the right internal jugular vein. Ultrasound guidance should include confirmation of the needle position in the vein before approaching the guide wire (short-axis/out-of-plane view (**a**) and long-axis/in-plane view (**b**)). In addition, the correct position of the guide wire in the vein (short-axis (**c**) and long-axis (**d**)) and the correct position of the catheter in the vein (short-axis (**e**) and long-axis (**f**)) should be confirmed

The use of real-time US then allows confirmation that the needle tip is placed centrally in the vein before approaching the guide wire (Fig. 6a, b).

### Confirm wire position in the vein

As a next step after wire advancement, the correct position of the guide wire should be confirmed in both a short-axis and a long-axis US view (Fig. 6c, d).

### Confirm catheter position in the vein

Finally, after placement of the CVC over the guide wire, the correct position of the CVC in the vein can be visualized with US, again in a short-axis and a long-axis view (Fig. 6e, f).

**Fig. 7:**
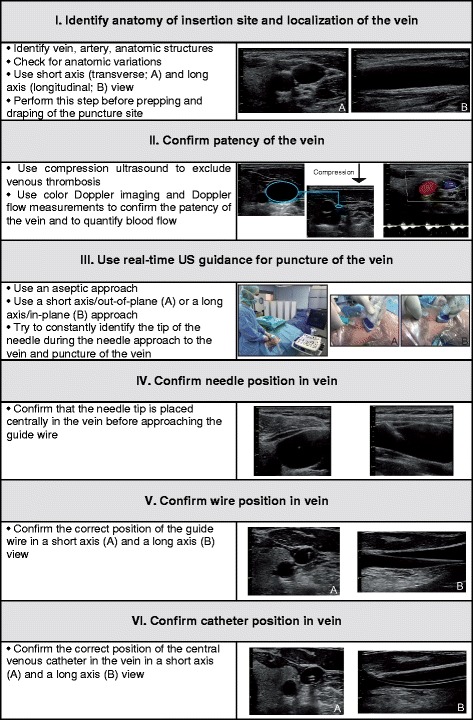
Six-step approach to ultrasound-guided central venous catheter placement

Figure 7 summarizes the six-step approach to US-guided CVC insertion.

## How to integrate knowledge from landmark and ultrasound techniques?

To achieve the personal best skill level for CVC placement, it is crucial that one combines and integrates the anatomic knowledge from landmark techniques and the knowledge gained from US-guided vascular access (knowledge about image display and converting the 2D image into 3D reality, and hand–eye coordination) [31]. In this context, previous US studies provided important information on the effect of different interventions on the venous puncture sites.

It has been demonstrated repeatedly that positioning of the patient in a head-down (Trendelenburg) position increases the filling and thus the cross-sectional lumen of the IJV [33]. On the contrary, to increase the lumen of the FV, patients can be positioned in a head-up (reverse Trendelenburg) position [34]. Positioning of the leg in an abducted and externally rotated position also can help to maximize the cross-sectional diameter of the FV [35].

For the IJV, imaging studies showed that the position of the head plays an important role in optimizing the conditions during CVC placement. Several studies demonstrated that rotation of the head to the opposite side increases the overlap of the IJV and the carotid artery [36–38]. In a US study, Miki et al. [37] investigated in 30 volunteers the anatomical relationship between the IJV and the carotid artery during head rotation. The overlap of the IJV and the carotid artery gradually increased with increasing rotation of the head to the left. In parallel, however, the flattening of the IJV decreased with head rotation to the left. DeAngelis et al. [39] described that the IJV becomes more vertically separated from the carotid artery at more extreme angles of contralateral head rotation. These findings underline that US should be used in each individual patient to assess the optimal angle of head rotation and best approach to the IJV.

## Technical developments in the field of ultrasound-guided vascular access

Needle guides are devices placed on the US transducer that might improve the cannulation success by facilitating aligning the angle between the US and the needle plane so that the two planes intersect at the depth of the vessel selected for cannulation [40]. Especially for inexperienced users [41], the needle guides help to guide the needle along the path of the US beam at the correct angle and distance depending on the depth of the targeted structure. Needle guides facilitate faster cannulation for IJV CVCs (only for inexperienced operators) [42] and SV CVCs [40]. Nevertheless, in a simulation model study, a needle guide used in a long-axis vessel approach improved needle visualization but did not improve puncture of the target vessel compared with a free-hand technique [43]. Altogether, based on the contradicting evidence [43, 44], no rigorous conclusion about the clinical value of needle guide devices can currently be drawn.

Different real-time 3D techniques (sometimes referred to as 4D US with time being the fourth dimension) for US-guided CVC placement have been described [45, 46]. Lower image resolution, larger US probe dimensions, and artifacts making needle visualization difficult, however, are still major limitations of this innovative concept [46].

## Limitations of ultrasound-guided central venous catheter placement

Although US is noninvasive and thus does not bear a risk to directly harm the patient, some limitations and disadvantages of US during central venous access are worth considering.

One might argue that the risk of catheter-related bloodstream infections might be higher if US is used for CVC placement without applying a strict aseptic approach as already described [47]. In addition, an insufficient number of US machines in a certain unit (intensive care unit or anesthesia induction area) might cause procedural delays [47]. Moreover, it is expensive to purchase and maintain US machines and to provide adequate training for all operators involved in CVC placement [47].

US might give the inexperienced user a false sense of security and mislead him/her to neglect traditionally taught principles with regard to needle direction. It is key to visualize the needle (or needle tip) constantly during needle advancement to avoid accidental arterial puncture, posterior wall penetration, or pneumothorax. In addition, rapid movements with the needle during "searching the needle on the US screen" must be avoided rigorously. To overcome these problems related to insufficient US skills and to ensure high-quality care, formal education and training (including simulation) with a structured certification of US skills for vascular access and the development of a consensus standard for these training programs has been suggested [13].

Moreover, concerns have been expressed that routine US use will result in a "de-skilling" with regard to the landmark techniques because these techniques will not be taught and practiced anymore, thus resulting in higher complication rates when CVCs need to be placed when US is not available (e.g., in emergencies) [47].

Besides these general limitations, different problems specific for the different anatomical sites for CVC placement might occur during US-guided CVC placement. In patients with a shorter neck anatomy, the long-axis US view of the IJV might be difficult to obtain. Although the FV can usually be visualized easily using US in adults, in severely obese patients a second operator might be necessary to provide access to the inguinal region. In addition, a curved-array abdominal US probe can be necessary for visualizing deeper anatomic structures. In comparison to the IJV and FV, the anatomic location and course of the SV under the clavicle bone can be more difficult to visualize using US. Smaller US probes can facilitate US-guided access to the SV [48, 49]. Of note, the use of US to puncture the SV results in a puncture site that is usually more lateral compared to the landmark puncture technique. The close proximity of the vessels and the pleura must be kept in mind also during US-guided puncture of the SV. Because the angle of cannulation is usually steeper when using US, it is especially important to align and constantly visualize the needle to avoid pleural injury.

## Conclusion

US guidance can improve patient safety and procedural quality during CVC placement in the IJV, FV, and SV. Based on evidence from clinical studies, several guidelines of medical societies strongly recommend the use of US for CVC placement in the IJV. Data from survey studies show that there is still a gap between the existing evidence and guidelines and the use of US in clinical practice. We recommend a six-step systematic approach for US-guided central venous access. To achieve the best skill level for CVC placement the knowledge from anatomic landmark techniques and the knowledge from US-guided CVC placement need to be combined and integrated.

## Abbreviations

CVC: Central venous catheter
FV: Femoral vein
IJV: Internal jugular vein
SV: Subclavian vein
US: Ultrasound

## References

[1] McGee DC, Gould MK (2003). Preventing complications of central venous catheterization. N Engl J Med..

[2] Merrer J, De Jonghe B, Golliot F, Lefrant JY, Raffy B, Barre E, Rigaud JP, Casciani D, Misset B, Bosquet C, Outin H, Brun-Buisson C, Nitenberg G (2001). Complications of femoral and subclavian venous catheterization in critically ill patients: a randomized controlled trial. JAMA..

[3] Hoffman T, Du Plessis M, Prekupec MP, Gielecki J, Zurada A, Shane Tubbs R, Loukas M (2017). Ultrasound-guided central venous catheterization: a review of the relevant anatomy, technique, complications, and anatomical variations. Clin Anat..

[4] Gordon AC, Saliken JC, Johns D, Owen R, Gray RR (1998). US-guided puncture of the internal jugular vein: complications and anatomic considerations. J Vasc Interv Radiol..

[5] Turba UC, Uflacker R, Hannegan C, Selby JB (2005). Anatomic relationship of the internal jugular vein and the common carotid artery applied to percutaneous transjugular procedures. Cardiovasc Intervent Radiol..

[6] Denys BG, Uretsky BF (1991). Anatomical variations of internal jugular vein location: impact on central venous access. Crit Care Med..

[7] Benter T, Teichgraber UK, Kluhs L, Papadopoulos S, Kohne CH, Felix R, Dorken B (2001). Anatomical variations in the internal jugular veins of cancer patients affecting central venous access. Anatomical variation of the internal jugular vein. Ultraschall Med.

[8] Docktor B, So CB, Saliken JC, Gray RR (1996). Ultrasound monitoring in cannulation of the internal jugular vein: anatomic and technical considerations. Can Assoc Radiol J..

[9] Jenssen C, Brkljacic B, Hocke M, Ignee A, Piscaglia F, Radzina M, Sidhu PS, Dietrich CF (2016). EFSUMB Guidelines on Interventional Ultrasound (INVUS). Part VI—Ultrasound-Guided Vascular Interventions. Ultraschall Med..

[10] Buchanan MS, Backlund B, Liao MM, Sun J, Cydulka RK, Smith-Coggins R, Kendall J (2014). Use of ultrasound guidance for central venous catheter placement: survey from the American Board of Emergency Medicine Longitudinal Study of Emergency Physicians. Acad Emerg Med..

[11] Beaudoin FL, Merchant RC, Lincoln J, Gardiner F, Liebmann O, Cohn J (2011). Bedside ultrasonography detects significant femoral vessel overlap: implications for central venous cannulation. CJEM..

[12] Hilty WM, Hudson PA, Levitt MA, Hall JB (1997). Real-time ultrasound-guided femoral vein catheterization during cardiopulmonary resuscitation. Ann Emerg Med..

[13] Lamperti M, Bodenham AR, Pittiruti M, Blaivas M, Augoustides JG, Elbarbary M, Pirotte T, Karakitsos D, Ledonne J, Doniger S, Scoppettuolo G, Feller-Kopman D, Schummer W, Biffi R, Desruennes E, Melniker LA, Verghese ST (2012). International evidence-based recommendations on ultrasound-guided vascular access. Intensive Care Med..

[14] Dietrich CF, Horn R, Morf S, Chiorean L, Dong Y, Cui XW, Atkinson NS, Jenssen C (2016). Ultrasound-guided central vascular interventions, comments on the European Federation of Societies for Ultrasound in Medicine and Biology guidelines on interventional ultrasound. J Thorac Dis..

[15] Troianos CA, Hartman GS, Glas KE, Skubas NJ, Eberhardt RT, Walker JD, Reeves ST (2012). Special articles: guidelines for performing ultrasound guided vascular cannulation: recommendations of the American Society of Echocardiography and the Society Of Cardiovascular Anesthesiologists. Anesth Analg..

[16] Blaivas M, Brannam L, Fernandez E (2003). Short-axis versus long-axis approaches for teaching ultrasound-guided vascular access on a new inanimate model. Acad Emerg Med..

[17] Chittoodan S, Breen D, O'Donnell BD, Iohom G (2011). Long versus short axis ultrasound guided approach for internal jugular vein cannulation: a prospective randomised controlled trial. Med Ultrason..

[18] Vezzani A, Manca T, Brusasco C, Santori G, Cantadori L, Ramelli A, Gonzi G, Nicolini F, Gherli T, Corradi F. A randomized clinical trial of ultrasound-guided infra-clavicular cannulation of the subclavian vein in cardiac surgical patients: short-axis versus long-axis approach. Intensive Care Med. 2017. doi: 10.1007/s00134-017-4756-6. [Epub ahead of print] 10.1007/s00134-017-4756-6 28289815

[19] Stone MB, Moon C, Sutijono D, Blaivas M (2010). Needle tip visualization during ultrasound-guided vascular access: short-axis vs long-axis approach. Am J Emerg Med..

[20] Phelan M, Hagerty D (2009). The oblique view: an alternative approach for ultrasound-guided central line placement. J Emerg Med..

[21] Wilson JG, Berona KM, Stein JC, Wang R (2014). Oblique-axis vs. short-axis view in ultrasound-guided central venous catheterization. J Emerg Med.

[22] Brass P, Hellmich M, Kolodziej L, Schick G, Smith AF (2015). Ultrasound guidance versus anatomical landmarks for internal jugular vein catheterization. Cochrane Database Syst Rev.

[23] Brass P, Hellmich M, Kolodziej L, Schick G, Smith AF (2015). Ultrasound guidance versus anatomical landmarks for subclavian or femoral vein catheterization. Cochrane Database Syst Rev.

[24] Lalu MM, Fayad A, Ahmed O, Bryson GL, Fergusson DA, Barron CC, Sullivan P, Thompson C (2015). Ultrasound-guided subclavian vein catheterization: a systematic review and meta-analysis. Crit Care Med..

[25] Rupp SM, Apfelbaum JL, Blitt C, Caplan RA, Connis RT, Domino KB, Fleisher LA, Grant S, Mark JB, Morray JP, Nickinovich DG, Tung A (2012). Practice guidelines for central venous access: a report by the American Society of Anesthesiologists Task Force on Central Venous Access. Anesthesiology..

[26] Frankel HL, Kirkpatrick AW, Elbarbary M, Blaivas M, Desai H, Evans D, Summerfield DT, Slonim A, Breitkreutz R, Price S, Marik PE, Talmor D, Levitov A (2015). Guidelines for the Appropriate Use of Bedside General and Cardiac Ultrasonography in the Evaluation of Critically Ill Patients—Part I: General Ultrasonography. Crit Care Med..

[27] Bodenham Chair A, Babu S, Bennett J, Binks R, Fee P, Fox B, Johnston AJ, Klein AA, Langton JA, McLure H, Tighe SQ (2016). Association of Anaesthetists of Great Britain and Ireland: safe vascular access 2016. Anaesthesia..

[28] McGrattan T, Duffty J, Green JS, O'Donnell N (2008). A survey of the use of ultrasound guidance in internal jugular venous cannulation. Anaesthesia..

[29] Soni NJ, Reyes LF, Keyt H, Arango A, Gelfond JA, Peters JI, Levine SM, Adams SG, Restrepo MI (2016). Use of ultrasound guidance for central venous catheterization: a national survey of intensivists and hospitalists. J Crit Care..

[30] Maizel J, Bastide MA, Richecoeur J, Frenoy E, Lemaire C, Sauneuf B, Dupont H, Tamion F, Nseir S, Du Cheyron D (2016). Practice of ultrasound-guided central venous catheter technique by the French intensivists: a survey from the BoReal study group. Ann Intensive Care..

[31] Weiner MM, Geldard P, Mittnacht AJ (2013). Ultrasound-guided vascular access: a comprehensive review. J Cardiothorac Vasc Anesth..

[32] Marhofer P, Fritsch G (2015). Sterile working in ultrasonography: the use of dedicated ultrasound covers and sterile ultrasound gel. Expert Rev Med Devices..

[33] Mallory DL, Shawker T, Evans RG, McGee WT, Brenner M, Parker M, Morrison G, Mohler P, Veremakis C, Parrillo JE (1990). Effects of clinical maneuvers on sonographically determined internal jugular vein size during venous cannulation. Crit Care Med..

[34] Stone MB, Price DD, Anderson BS (2006). Ultrasonographic investigation of the effect of reverse Trendelenburg on the cross-sectional area of the femoral vein. J Emerg Med..

[35] Randall C, Schmeiser E, Fiers E, Little A, Dogbey G, Richardson G (2014). Ultrasound investigation of leg position to enhance femoral vein exposure for cannulation. J Emerg Med..

[36] Sulek CA, Gravenstein N, Blackshear RH, Weiss L (1996). Head rotation during internal jugular vein cannulation and the risk of carotid artery puncture. Anesth Analg..

[37] Miki I, Murata S, Nakazawa K, Onozawa S, Mine T, Ueda T, Yamaguchi H, Yasui D, Takeda M, Kumita S (2014). Anatomical relationship between the common carotid artery and the internal jugular vein during head rotation. Ultrasound..

[38] Troianos CA, Kuwik RJ, Pasqual JR, Lim AJ, Odasso DP (1996). Internal jugular vein and carotid artery anatomic relation as determined by ultrasonography. Anesthesiology..

[39] DeAngelis V, Denny J, Chyu D, Jan T, Lemaire A, Chiricolo A, Solina A (2015). The optimal angle of head rotation for internal jugular cannulation as determined by ultrasound evaluation. J Cardiothorac Vasc Anesth..

[40] Maecken T, Heite L, Wolf B, Zahn PK, Litz RJ (2015). Ultrasound-guided catheterisation of the subclavian vein: freehand vs needle-guided technique. Anaesthesia..

[41] Jaffer U, Normahani P, Singh P, Aslam M, Standfield NJ (2015). Randomized study of teaching ultrasound-guided vascular cannulation using a phantom and the freehand versus needle guide-assisted puncture techniques. J Clin Ultrasound..

[42] Augoustides JG, Horak J, Ochroch AE, Vernick WJ, Gambone AJ, Weiner J, Pinchasik D, Kowalchuk D, Savino JS, Jobes DR (2005). A randomized controlled clinical trial of real-time needle-guided ultrasound for internal jugular venous cannulation in a large university anesthesia department. J Cardiothorac Vasc Anesth..

[43] Ball RD, Scouras NE, Orebaugh S, Wilde J, Sakai T (2012). Randomized, prospective, observational simulation study comparing residents' needle-guided vs free-hand ultrasound techniques for central venous catheter access. Br J Anaesth..

[44] Luyet C, Hartwich V, Urwyler N, Schumacher PM, Eichenberger U, Vogt A (2011). Evaluation of a novel needle guide for ultrasound-guided phantom vessel cannulation. Anaesthesia..

[45] Dowling M, Jlala HA, Hardman JG, Bedforth NM (2011). Real-time three-dimensional ultrasound-guided central venous catheter placement. Anesth Analg..

[46] French JL, Raine-Fenning NJ, Hardman JG, Bedforth NM (2008). Pitfalls of ultrasound guided vascular access: the use of three/four-dimensional ultrasound. Anaesthesia..

[47] Hessel EA (2009). Con: we should not enforce the use of ultrasound as a standard of care for obtaining central venous access. J Cardiothorac Vasc Anesth..

[48] Kim SC, Graff I, Sommer A, Hoeft A, Weber S (2016). Ultrasound-guided supraclavicular central venous catheter tip positioning via the right subclavian vein using a microconvex probe. J Vasc Access..

[49] Lanspa MJ, Fair J, Hirshberg EL, Grissom CK, Brown SM (2014). Ultrasound-guided subclavian vein cannulation using a micro-convex ultrasound probe. Ann Am Thorac Soc..

